# SRSF3 and HNRNPH1 Regulate Radiation-Induced Alternative Splicing of Protein Arginine Methyltransferase 5 in Hepatocellular Carcinoma

**DOI:** 10.3390/ijms232314832

**Published:** 2022-11-27

**Authors:** Chaowei Wen, Zhujun Tian, Lan Li, Tongke Chen, Huajian Chen, Jichen Dai, Zhenzhen Liang, Shumei Ma, Xiaodong Liu

**Affiliations:** 1 School of Public Health and Management, Wenzhou Medical University, Wenzhou 325035, China; 2 School of Laboratory Medicine and Life Science, Wenzhou Medical University, Wenzhou 325035, China; 3 Laboratory Animal Center, Wenzhou Medical University, Wenzhou 325035, China; 4 School of the 2nd Clinical Medical Sciences, Wenzhou Medical University, Wenzhou 325035, China; 5 NHC Key Laboratory of Radiobiology, School of Public Health, Jilin University, Changchun 130021, China; 6 South Zhejiang Institute of Radiation Medicine and Nuclear Technology, Wenzhou 325014, China; 7 Key Laboratory of Watershed Science and Health of Zhejiang Province, Wenzhou 325035, China

**Keywords:** protein arginine methyltransferase 5, alternative splicing, SRSF3, HNRNPH1, HCC, ionizing radiation

## Abstract

Protein arginine methyltransferase 5 (PRMT5) is an epigenetic regulator which has been proven to be a potential target for cancer therapy. We observed that *PRMT5* underwent alternative splicing (AS) and generated a spliced isoform *PRMT5-ISO5* in hepatocellular carcinoma (HCC) patients after radiotherapy. However, the regulatory mechanism and the clinical implications of IR-induced *PRMT5* AS are unclear. This work revealed that serine and arginine rich splicing factor 3 (SRSF3) silencing increased *PRMT5-ISO5* level, whereas heterogeneous nuclear ribonucleoprotein H 1 (HNRNPH1) silencing reduced it. Then, we found that SRSF3 and HNRNPH1 competitively combined with *PRMT5* pre-mRNA located at the region around the 3′- splicing site on intron 2 and the alternative 3′- splicing site on exon 4. IR-induced SRSF3 downregulation led to an elevated level of *PRMT5-ISO5*, and exogenous expression of *PRMT5-ISO5* enhanced cell radiosensitivity. Finally, we confirmed in vivo that IR induced the increased level of *PRMT5-ISO5* which in turn enhanced tumor killing and regression, and liver-specific Prmt5 depletion reduced hepatic steatosis and delayed tumor progression of spontaneous HCC. In conclusion, our data uncover the competitive antagonistic interaction of SRSF3 and HNRNPH1 in regulating *PRMT5* splicing induced by IR, providing potentially effective radiotherapy by modulating *PRMT5* splicing against HCC.

## 1. Introduction

Hepatocellular carcinoma (HCC) is the fourth leading cause of cancer-related deaths worldwide and represents three-fourths of primary hepatic malignancies. Unfortunately, more than 70% of patients harboring a high tumor burden or liver dysfunction remain unsuitable for definitive resection [1]. Stereotactic body radiotherapy (SBRT) is a new noninvasive treatment for HCC patients who are not suitable for radical surgery [2], representing an effective method for controlling local recurrence and optimizing surgical strategies [3,4]. However, evidence on the molecular mechanism of SBRT in HCC patients is still lacking.

Alternative splicing (AS) of RNA is a posttranscriptional process found in the majority of human multiexon genes. Differential AS events were predicted to be risk factors for HCC patient survival and were significantly enriched in metabolism-related pathways [5]. As a prognostic biomarker of HCC [6], protein arginine methyltransferase 5 (PRMT5) has been proven to be an essential epigenetic regulator for regulating cell proliferation and apoptosis by methylation of arginine residues [7]. Targeting PRMT5 might be an effective strategy for some hematological and solid tumors, however, there are still challenges to the clinical application of PRMT5 inhibitors due to safety and unavoidable toxicity. Currently, PRMT5 has been identified to generate different transcripts with unequal biological activities. The canonical transcript *PRMT5* isoform a (NM_006109.5, *PRMT5-ISO1*) translates into full-length protein, whereas another transcript *PRMT5* isoform e (NM_001282955.2, *PRMT5-ISO5*) generates truncated protein by skipping exon 3 and part of exon 4. When compared to full-length PRMT5, the truncated protein can exhibit distinct localization and preferential control of critical genes for cell cycle arrest [8]. PRMT5 mediates radiotherapy resistance in prostate cancer [9], implying that targeting *PRMT5* splicing may be an effective radiosensitization strategy for cancer radiotherapy.

Based on the above consideration, we focus on the molecular mechanism and preclinical evaluation of IR-induced *PRMT5* splicing. In vitro cell experiments mainly explored that (1) IR induced *PRMT5* AS by changing the levels of serine/arginine-rich splicing factor 3 (SRSF3) and heterogeneous nuclear ribonucleoprotein H 1 (HNRNPH1); (2) the interaction of SRSF3 and HNRNPH1 involved in the process of *PRMT5* splicing; (3) the elevated level of *PRMT5-ISO5* sensitized HCC cells to radiation. The clinical and preclinical studies mainly involved the analysis of *PRMT5-ISO5* levels responded to radiation, the effects of increased *PRMT-ISO5* and PRMT5 deficiency on xenograft tumor and primary HCC, by establishing xenograft tumor models, and liver-specific Prmt5 knockout spontaneous HCC mice. Considering the increased incidence and death rates of HCC, we hope modulating *PRMT5* splicing will be an effective choice to enhance the radiotherapeutic effect on HCC.

## 2. Results

### 2.1. Elevated Levels of PRMT5-ISO5 Improve the Poor Prognosis of HCC

**Figure 1:**
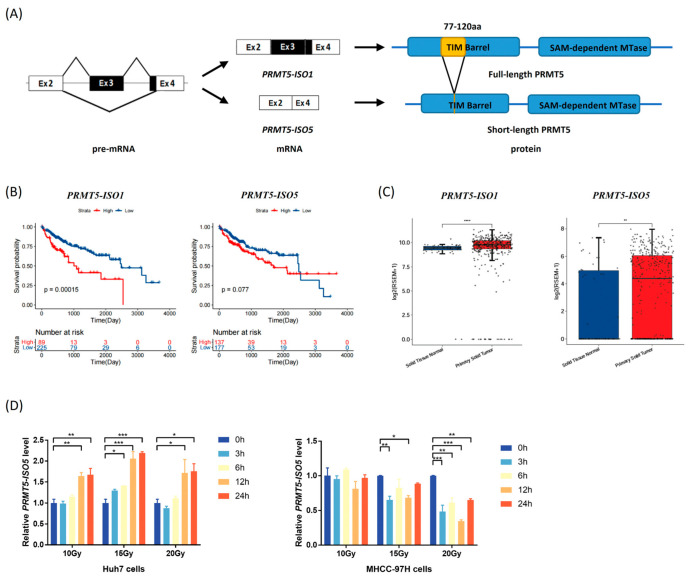
Radiation-induced *PRMT5-ISO5* production improves the poor prognosis of HCC. (**A**) The splicing variant *PRMT5-ISO5* skips exon 3 and part of exon 4 of *PRMT5* and forms the shorter protein. (**B**) The Kaplan–Meier curves of overall survival were analyzed using log-rank tests. The patient datasets were from the TSVdb TCGA Splicing Variants Database (http://www.tsvdb.com/plot.html, accessed on 14 September 2021). (**C**) The transcriptional levels of *PRMT5* splicing variants associated with HCC were analyzed using the Kruskal–Wallis test. ** *p* < 0.01, **** *p* < 0.0001. (**D**) The different changes in *PRMT5-ISO5* levels induced by IR were detected by RT–qPCR. * *p* < 0.05, ** *p* < 0.01, *** *p* < 0.001.

Human *PRMT5* precursor mRNA (pre-mRNA) can be alternatively spliced into different splicing isoforms. The splice variant *PRMT5-ISO5* lacks exon 3 and part of exon 4 (Figure 1A), resulting in a truncated protein with a shortened TIM domain. Bioinformatics analysis revealed that HCC patients with higher *PRMT5-ISO1* levels survived for significantly shorter times, whereas higher *PRMT5-ISO5* levels improved HCC patients’ poor prognosis (Figure 1B). Although both transcripts were found to be overexpressed in tumors, the expression distribution of *PRMT5* isoforms in HCC suggested that decreasing *PRMT5-ISO1* and increasing *PRMT5-ISO5* might be beneficial to HCC patients (Figure 1C and Figure S1A). Next, we discovered that *PRMT5-ISO1* decreased while *PRMT5-ISO5* increased in blood samples from HCC patients receiving SBRT (Figure S1B). These findings indicated that increasing *PRMT5-ISO5* levels may be beneficial in HCC radiotherapy.

To confirm that IR could increase the *PRMT5-ISO5* transcript level, three HCC cell lines (Huh7, HepG2, and MHCC-97H cells) were treated with IR (Figure S1C). By performing the RT-qPCR assay, we found that *PRMT5-ISO5* levels in Huh7 cells increased steadily and continuously after IR treatment (Figure 1D). However, *PRMT5-ISO5* slightly decreased in MHCC-97H cells, and mildly decreased and then recovered in HepG2 cells treated with a higher dose of IR (Figure 1D and Figure S1D). We were unable to detect truncated PRMT5 which is translated from *PRMT5-ISO5*, partly due to the limited specificity of the antibody that targeted PRMT5. This finding supported the notion that increasing *PRMT5-ISO5* levels could, at least in part, improve the poor prognosis of HCC patients.

### 2.2. IR induces PRMT5-ISO5 Transcript Levels by Changing the Levels of SRSF3 and HNRNPH1

**Figure 2:**
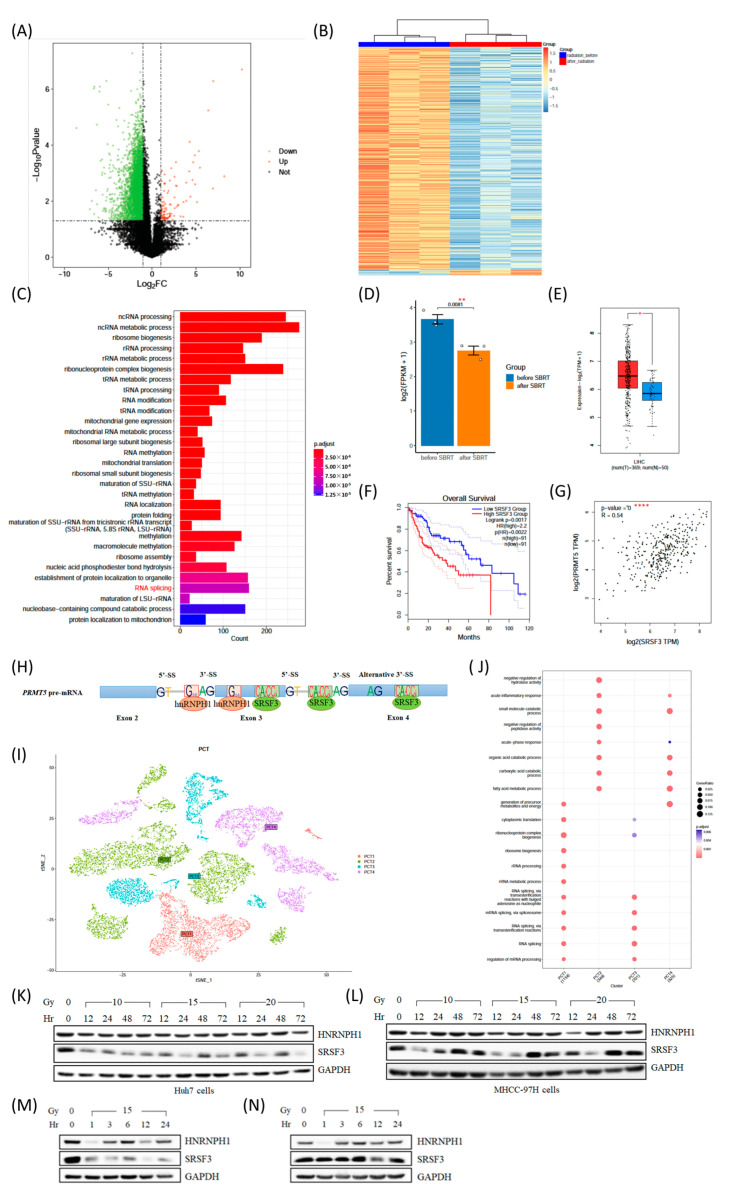
IR-induced *PRMT5-ISO5* production is regulated by SRSF3 and HNRNPH1 in HCC cells. (**A**) Significantly differential expression of genes is shown in a volcano plot. (**B**) Clustering analysis is shown in the heatmap. (**C**) GO analysis was performed, and the top 30 enriched pathways are shown in bar charts. (**D**) The expression level of SRSF3 in HCC patients who received SBRT (n = 3). (**E**,**F**) Bioinformatics analysis of LIHC patients and normal controls based on GEPIA2. (**E**) The differential expression of SRSF3. (**F**) The overall survival analysis diagram of SRSF3. (**G**) The correlation analysis of PRMT5 and SRSF3. * *p* < 0.05, ** *p* < 0.01, **** *p* < 0.0001. (**H**) The predicted RNA target regions and motifs bound by SRSF3 and HNRNPH1. (**I**) t-SNE of 4 cell populations from scRNA-seq datasets (GSE149614). (**J**) GO enrichment analysis of the 4 cell populations. (**K**–**N**) The expression level of SRSF3 and HNRNPH1 in Huh7 and MHCC-97H cells induced by IR at different time points.

Subsequently, we analyzed RNA-seq data from HCC patients receiving SBRT and found that the vast majority of genes were downregulated following radiation treatment (Figure 2A,B). Further exploration revealed that genes with significant changes were enriched in the RNA splicing pathway, which included dozens of representative splicing factors (Figure 2C). Subsequently, we evaluated SR family gene expression and found that SRSF2, 3, 5, 6, and 11 had significantly lower levels of expression after radiation treatment (Figure 2D and Figure S2A). Among these downregulated SRSFs, SRSF2, 3, and 11 were markedly increased in HCC tissues and correlated with poor patient outcomes. Furthermore, SRSF2 and 3 showed the strongest correlation with *PRMT5* in LIHC tumor specimens (Figure 2E–G and Figure S2B–D). As the oncogenic activity and target AS events of SRSF2 have been studied profoundly, we finally focused on SRSF3, which has not been investigated for its regulatory actions in radiotherapy for HCC.

Furthermore, we also showed that HNRNPH1 was linked to the methyltransferase function of PRMT5, which was dependent on its binding to methylosome protein 50 (MEP50) (Figure S2E) and predicted to be the adjacent position at which SRSF3 binds to *PRMT5* pre-mRNA using SpliceAid 2 [10] (Figure 2H). Given the foregoing, we further investigated the interaction of SRSF3 and HNRNPH1 in HCC patients. By analyzing the scRNA-seq dataset (GSE149614), more than 70,000 single-cell transcriptomes from 10 HCC patients were obtained. After standardization and dimensionality reduction and clustering, 15 clusters of cells derived from epithelial cells were defined using the clusterProfiler package based on the transcriptional levels of SRSF3 and HNRNPH1 (Figure S2F–I). Specifically, genes with significant changes from two cell populations (PCT1 and PCT3) that were identified with high levels of SRSF3 were enriched in the RNA splicing pathway (Figure 2I–J). These findings suggested that, along with HNRNPH1, SRSF3 might play a dominant role in regulating HCC-related AS events.

With the assumption that SRSF3 and HNRNPH1 may be the major effectors in IR-induced *PRMT5* splicing, we observed SRSF3 downregulation, particularly a continuous decrease in Huh7 cells after IR treatment (Figure 2K–N and Figure S2J). However, IR barely induced an instantaneous decrease in HNRNPH1 (Figure 2K–N and Figure S2J). Moreover, the *PRMT5-ISO5* transcript level changed within 24 h after IR treatment (Figure S2K). Together, these results indicated that IR-induced SRSF3 downregulation might disrupt the interaction of HNRNPH1 with *PRMT5* pre-mRNA and have a major effect on regulating *PRMT5-ISO5* production.

### 2.3. The Antagonistic Interaction of SRSF3 and HNRNPH1 Depends on Their Competitive Binding with PRMT5

**Figure 3:**
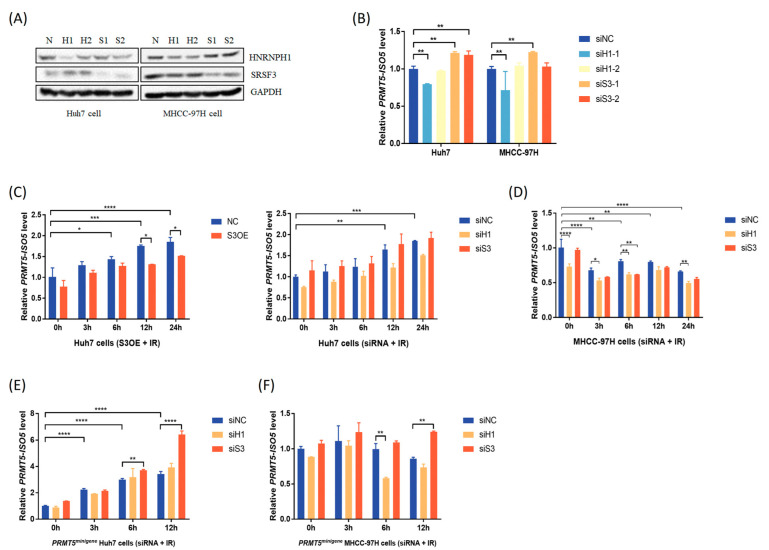
SRSF3 and HNRNPH1 play opposite roles in *PRMT5-ISO5* production induced by IR. (**A**) The efficiencies of HNRNPH1 silencing or SRSF3 depletion. H1/2: si-*HNRNPH1*-1/2, S1/2: si-*SRSF3*-1/2; N: nonsense siRNA. (**B**) The transcriptional level of *PRMT5-ISO5* induced by HNRNPH1 silencing or SRSF3 depletion (siH1-1/2: si-*HNRNPH1*-1/2, siS3-1/2: si-*SRSF3*-1/2; siNC: nonsense siRNA). (**C**,**D**) The transcriptional level of *PRMT5-ISO5* with different treatments in cells. siH1: si-*HNRNPH1*, siS3: si-*SRSF3*; siNC: nonsense siRNA; NC: vector; S3OE: SRSF3 overexpression. (**E**,**F**) The transcriptional level of *PRMT5-ISO5* with different treatments in cells. siH1: si-*HNRNPH1*, siS3: si-*SRSF3*; siNC: nonsense siRNA; *PRMT5*^minigene^: PRMT5 KO clones transfected with *PRMT5-minigene* construct. * *p* < 0.05, ** *p* < 0.01, *** *p* < 0.001, **** *p* < 0.0001.

To evaluate the functions of SRSF3 and HNRNPH1 on *PRMT5* splicing, wild-type Huh7 and MHCC-97H cells were transfected with si-*HNRNPH1* or si-*SRSF3* (Figure 3A and Figure S3A,B). We observed that HNRNPH1 depletion caused a decrease in *PRMT5-ISO5* levels, while SRSF3 silencing resulted in an increase in *PRMT5-ISO5* levels (Figure 3B). This result showed that SRSF3 and HNRNPH1 play opposite roles in regulating *PRMT5* splicing and *PRMT5-ISO5* production. Furthermore, SRSF3 overexpression significantly inhibited *PRMT5-ISO5* increase in Huh7 cells induced by IR (Figure 3C). Although HNRNPH1 silencing slightly reduced IR-induced *PRMT5-ISO5* levels, at least in part, there was no significant difference when compared to the control (Figure 3C,D). This result indicated that IR caused a more efficient change in *PRMT5-ISO5* levels than SRSF3 or HNRNPH1 interference in HCC cells. Interestingly, SRSF3 expression seemed to be more affected by IR treatment in Huh7 cells, whereas HNRNPH1 interference was more effective in rescuing *PRMT5-ISO5* downregulation in MHCC-97H cells treated with IR. Together, these results supported that SRSF3 and HNRNPH1 antagonistically regulated the splicing of *PRMT5*, and SRSF3 recovery could reverse the increase in *PRMT5-ISO5* levels induced by IR.

Regarding other effective factors, such as *PRMT5-AS1* (an antisense transcript of *PRMT5*) [11], we generated multiple single PRMT5 KO clones in the Huh7 and MHCC-97H cell lines using the CRISPR/Cas9 system and serial dilution method (Figure S3C). Despite screening for ~100 single clones of each cell line, we rarely observed complete *PRMT5* depletion at the protein level, but the transcriptional levels of *PRMT5*-minigene achieved dozens of times the residual expression of endogenous *PRMT5* mRNA (Figure S3D). Subsequently, cotransfection of the *PRMT5*-minigene construct and si-*HNRNPH1* or si-*SRSF3* was performed in PRMT5 KO clones to simulate *PRMT5* splicing. We found that IR still resulted in an increase in *PRMT5-ISO5* levels in *PRMT5^minigene^* Huh7 cells, which was enhanced by SRSF3 silencing (Figure 3E). Meanwhile, the IR-induced *PRMT5-ISO5* decrease was limited in *PRMT5^minigene^* MHCC-97H cells, although SRSF3 or HNRNPH1 silencing had no effects on *PRMT5* splicing (Figure 3F). These results indicated that other interferences, such as *PRMT5-AS1*, which relied on the 3′-terminal sequences of *PRMT5* pre-mRNA might contribute to the level change in *PRMT5-ISO5* induced by IR and should be investigated further.

**Figure 4:**
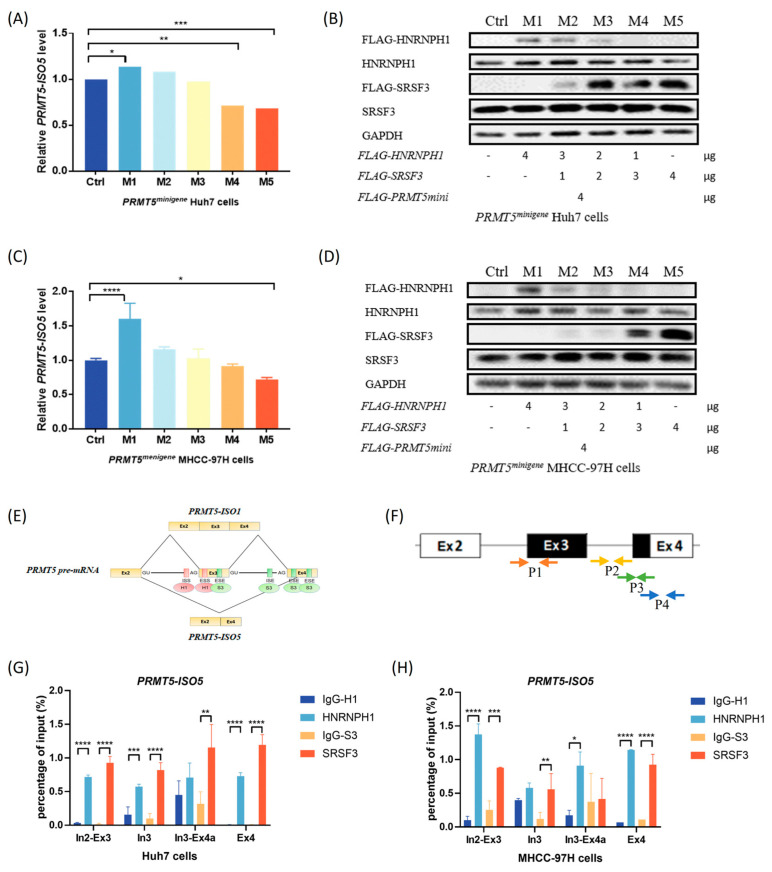
SRSF3 and HNRNPH1 competitively bind to *PRMT5* pre-mRNA and regulate *PRMT5* splicing. (**A**) The transcriptional level of *PRMT5-ISO5* in Huh7 cells post-treatment. (**B**) The endo- and exogenous expression level of HNRNPH1 and SRSF3 in Huh7 cells post-treatment. Ctrl: cells transfected with 4 μg pcDNA3.1-*FLAG* vector; M1-5: cells transfected with 4, 3, 2, 1, 0 μg pcDNA3.1-*FLAG-HNRNPH1* mixed with 0, 1, 2, 3, 4 μg pcDNA3.1-*FLAG*-*SRSF3*. FLAG-HNRNPH1 and FLAG-SRSF3 fusion proteins (exogenous expression) were detected with anti-FLAG, while HNRNPH1, SRSF3, and GAPDH (endogenous expression) were determined using target antibodies. * *p* < 0.05, ** *p* < 0.01, *** *p* < 0.001, **** *p* < 0.0001. (**C**) The transcriptional level of *PRMT5-ISO5* in MHCC-97H cells post-treatment. (**D**) The endo- and exogenous expression level of HNRNPH1 and SRSF3 in MHCC-97H cells post-treatment. Ctrl: cells transfected with 4 μg pcDNA3.1-*FLAG* vector; M1-5: cells transfected with 4, 3, 2, 1, 0 μg pcDNA3.1-*FLAG-HNRNPH1* mixed with 0, 1, 2, 3, 4 μg pcDNA3.1-*FLAG*-*SRSF3*. FLAG-HNRNPH1 and FLAG-SRSF3 fusion proteins (exogenous expression) were detected with anti-FLAG, while HNRNPH1, SRSF3, and GAPDH (endogenous expression) were determined using target antibodies. * *p* < 0.05, ** *p* < 0.01, *** *p* < 0.001, **** *p* < 0.0001. (**E**) The potential binding region of HNRNPH1/SRSF3 in *PRMT5* pre-mRNA. (**F**) The primers targeting the potential binding region were designed for RIP-qPCR detection (P1: primers of pair 1 targeted to intron 2 and partial exon 3; P2: primers of pair 2 located in intron 3; P3: primers of pair 3 amplified for intron 3 and partial exon 4 (exon 4A); P4: primers of pair 4 targeted to exon 4). (**G**,**H**) The fold enrichment of SRSF3 or HNRNPH1 binds to the target region of *PRMT5* pre-mRNA (In2: intron 2; Ex3: exon 3; In3: intron 3; Ex4a: partial exon 4 (exon 4A); Ex4: exon 4). IgG-H1 and HNRNPH1 were collected from HNRNPH1-overexpressing cells, while IgG-S3 and SRSF3 were obtained from cells transfected with the pcDNA3.1-*FLAG-SRSF3* expression construct. Fold enrichment was determined after normalization to the input compared with the IgG control. * *p* < 0.05, ** *p* < 0.01, *** *p* < 0.001, **** *p* < 0.0001.

To verify the interaction of SRSF3 and HNRNPH1 in regulating *PRMT5* splicing, a mixture of the *SRSF3* construct and *HNRNPH1* construct was co-transfected into PRMT5 KO clones accompanied by *PRMT5-minigene* plasmid. We achieved a decrease in exogenous HNRNPH1 and a simultaneous increase in exogenous SRSF3 in the assay to investigate the level change in *PRMT5-ISO5*. In the results, we observed that exogenous HNRNPH1 downregulation and exogenous SRSF3 upregulation (Figure 4B,D) resulted in a switch from elevated levels of *PRMT5-ISO5* to decreased levels of *PRMT5-ISO5* (Figure 4A,C). In addition, the increase in *PRMT5-ISO5* appeared to be more sensitive to SRSF3 upregulation in *PRMT5^minigene^* Huh7 cells, whereas HNRNPH1 had a stronger effect in *PRMT5^minigene^* MHCC-97H cells (Figure 4A–D).

Taking into account the potential binding sites of SRSF3 and HNRNPH1 on *PRMT5* pre-mRNA from SpliceAid 2 [10], we designed four pairs of primers targeting the potential binding sites (Figure 4E,F). By performing a RIP-qPCR assay, we found that both SRSF3 and HNRNPH1 were significantly enriched in *PRMT5* pre-mRNA around the 3′ splicing site (3′ss) on intron 2 and the alternative 3′ss on exon 4 (Figure 4G,H). Furthermore, SRSF3 had a higher fold enrichment than HNRNPH1 in Huh7 cells (Figure 4G), whereas HNRNPH1 was preferentially enriched in the target region of *PRMT5* pre-mRNA in MHCC-97H cells (Figure 4H). This finding supported that the binding efficiencies of SRSF3 and HNRNPH1 to *PRMT5* pre-mRNA might influence the level change in *PRMT5-ISO5* response to IR treatment in different HCC cells.

### 2.4. Elevated Levels of PRMT5-ISO5 Suppress Cell Proliferation and Tumor Growth

**Figure 5:**
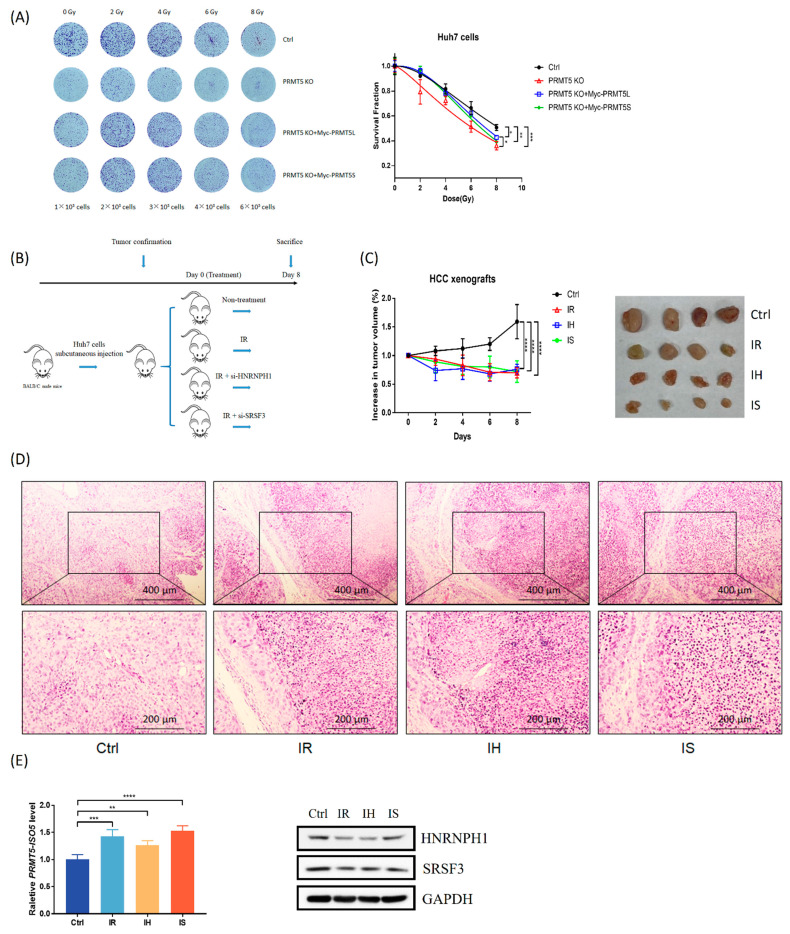
The increase in *PRMT5-ISO5* levels suppresses cell proliferation and inhibits tumor growth. (**A**) Left panel: the cell colony-forming abilities of cells treated with IR. Right panel: the survival fraction according to the calculation of colonies. Ctrl: Cas9-positive Huh7 cells; PRMT5 KO: PRMT5 KO Huh7 clones; PRMT5 KO + Myc-PRMT5L: PRMT5 KO Huh7 clones with exogenous expression of full-length PRMT5; PRMT5 KO + Myc-PRMT5S: PRMT5 KO Huh7 clones with exogenous expression of truncated PRMT5. (**B**) The procedure of the HCC xenograft model and treatments (n = 4 for each group). (**C**) Left panel: the volume of HCC xenograft tumors after treatment was normalized by the control group (n = 4 in each group). Right panel: tumors dissected from mice at the 8th day post-treatment. (**D**) The morphological change in HCC xenograft tumors at the 8th day post-treatment (100× and 200×). (**E**) Left panel: *PRMT5-ISO5* transcriptional level of HCC xenograft tumors at the 8th day post-treatment (n = 4 in each group). Right panel: HNRNPH1 and SRSF3 expression level of HCC xenograft tumors at the 8th day post-treatment (n = 4 in each group). Ctrl: HCC xenograft tumors without further treatment; IR: HCC xenograft tumors with IR treatment; IH: HCC xenograft tumors with IR treatment and in vivo si-HNRNPH1 intratumoral injection; IS: HCC xenograft tumors with IR treatment and in vivo si-SRSF3 intratumoral injection. ** *p* < 0.01, *** *p* < 0.001, **** *p* < 0.0001.

To investigate the effects of PRMT5 protein or the truncated isoform on radiosensitivity, PRMT5 KO and isoform rescue assays were used in HCC cells. We observed that PRMT5 deficiency sensitized Huh7 cells to IR treatment by suppressing cell proliferation (Figure 5A). Although both exogenous expressions of full-length PRMT5 or truncated PRMT5 had inadequate effects on recovering cell colony-forming abilities, there was no significant difference between the recovery of truncated PRMT5 expression and PRMT5 deficiency (Figure 5A). Subsequently, we also evaluated the methyltransferase activity of PRMT5 by detecting the level of symmetric dimethylarginine (SDMA) modification in PRMT5 knockdown cells with PRMT5 isoforms rescue. In the results, we observed that rescue of truncated PRMT5 expression reduced the level of SDMA modification compared with full-length PRMT5 rescue (Figure S3E). These data supported that increasing truncated PRMT5 expression (or *PRMT5-ISO5* transcript levels) probably increases cellular radiosensitivity and influences the methyltransferase activity of PRMT5.

To evaluate the tumorigenic effect of IR combined with RNA interference, which was associated with the *PRMT5-ISO5* transcript, the HCC xenograft model was constructed via subcutaneous injection of Huh7 cells (Figure 5B). Eight days after treatment, we observed a significant reduction in tumor volume in the experimental groups compared to the control group (Figure 5C). There was no significant difference among the experimental groups, which could be attributed to an insufficient time to show the subsequent significance associated with prognosis. In addition, the untreated tumor tissue showed a large amount of interstitial fibrosis and presented a typical tumor cell morphology (Figure 5D). When compared to untreated tumor tissues, the treated tumor tissue showed more severe structural degeneration and cell necrosis (Figure 5D). In addition, we also found an increase in *PRMT5-ISO5* levels in all experimental groups (Figure 5E). Although HNRNPH1 silencing reversed the increase in *PRMT5-ISO5* levels caused by IR while SRSF3 knockdown reinforced it, there was no significant difference in tumor growth between the experimental groups (Figure 5E and Figure S4A), indicating the prognostic value of *PRMT5-ISO5* in HCC. Together, these results still suggested that IR inhibited tumor growth and caused regression of HCC xenografts partially due to the increase in *PRMT5-ISO5* levels.

### 2.5. Liver-Specific Knockout of Prmt5 Inhibits Hepatocarcinogenesis

**Figure 6:**
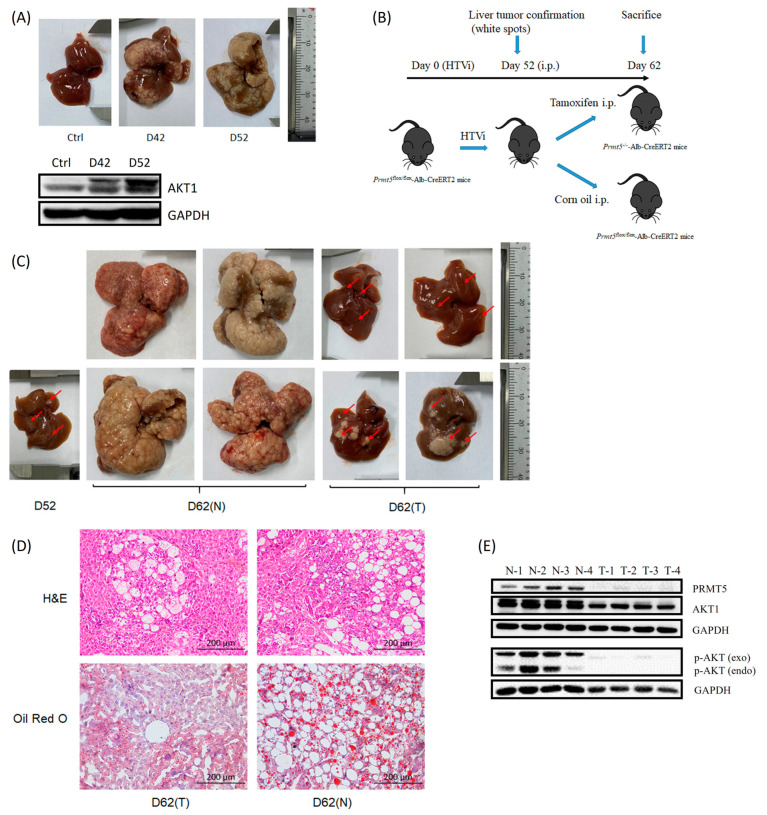
Liver-specific Prmt5 deficiency inhibits primary HCC progression. (**A**) Upper panel: livers dissected from the C57BL/6J mice untreated or treated with HTVi at the 42nd, or 52nd day post-treatment. Lower panel: the expression level of AKT1 of liver tissue or tumor from the mice. Ctrl: untreated controls; D42: HTVi treated mice at the 42nd day post-treatment; D52: HTVi treated mice at the 52nd day post-treatment. (**B**) The primary HCC model induced by overexpression of AKT1 and N-RasV12 using HTVi and liver-specific Prmt5 KO treatment (i.p.: intraperitoneal injection). (**C**) Livers dissected from the HTVi treated *Prmt5*^flox/flox^-Alb-CreERT2 mice with or without Prmt5 deficiency at the 52nd, or 62nd day post-HTVi treatment (white spots with red arrows represent spontaneous lesions). (**D**) The morphological change in liver tissue or tumor at the 62nd day post-HTVi treatment (200×). D: days after HTVi treatment; T: Prmt5 CKO group received tamoxifen intraperitoneal injection; N: non-CKO received corn oil intraperitoneal injection. (**E**) The expression level of PRMT5 and AKT1, and the phosphorylation of AKT at Ser473 both exogenously (exo) and endogenously (endo) of liver tissue or tumor from mice. N-1/4: non-CKO without tamoxifen injection; T-1/4: CKO group received tamoxifen injection.

To evaluate the effect of PRMT5 deficiency on primary HCC, we generated an autochthonous HCC model using the Akt/N-Ras-based HTVi method. By regularly sacrificing one cohort of C57BL/6J mice after HTVi, we found that exogenous Akt1 expression and the number of tumor nodules, and the size of the liver were increased with HCC progression (Figure 6A). In addition, H&E and Oil Red O staining of liver sections demonstrated vacuolar denaturation, inflammation, and intracellular lipid accumulation in the liver after HTVi treatment (Figure S4B). Furthermore, intraperitoneal injection of tamoxifen was performed in *Prmt5*^flox/flox^-Alb-CreERT2 mice to acquire liver-specific Prmt5 KO mice. We confirmed Prmt5 deficiency in liver tissues within continuous injection for 5 and 7 days, and negative or trace amounts of Prmt5 were detected 30 days after completing the injection (Figure S4C).

To confirm whether liver-specific Prmt5 deficiency could achieve remission of autochthonous HCC, Akt/N-Ras-based HTVi and tamoxifen-inducible deletion of Prmt5 specifically within the liver were used in *Prmt5*^flox/flox^-Alb-CreERT2 mice (Figure 6B). In this study, when tumors were visible on the 52nd day post-HTVi treatment, intraperitoneal injection of tamoxifen for 7 days was subsequently performed. On the 62nd day post-HTVi treatment, all the mice were sacrificed. The results showed a dramatic decrease in the number of tumor nodules visible on the surface of the liver from liver-specific Prmt5 KO mice (Figure 6C). Furthermore, H&E and Oil Red O staining of liver sections from liver-specific Prmt5 KO mice also showed reduced pathology associated with steatosis, hepatocyte bubble morphology, and inflammation in livers (Figure 6D). We also observed that the exogenous expression of Akt1 and the phosphorylation of Akt at Ser473 (exogenous and endogenous) were markedly reduced, along with Prmt5 deficiency (Figure 6E). Together, these data indicated that liver-specific Prmt5 deficiency prominently inhibits Akt/N-Ras-derived hepatocarcinogenesis partially by diminishing Akt1 phosphorylation, providing an exploration of PRMT5-related pathogenesis in primary HCC models.

## 3. Discussion

### 3.1. IR Induces the Generation of the PRMT5-ISO5 Transcript Which Improves the Poor Prognosis of HCC Patients

Pre-mRNA splicing is critical for the expression of more than 95% of human genes in posttranscriptional processes. Splicing regulation is not only essential for maintaining cellular homeostasis and cell development but is also associated with hallmarks of cancer. Cancer cells are vulnerable to splicing perturbations, leading to the exploitation of pharmacology and therapeutics. Recently, radiation has been proven to induce AS events, which may play a role in the radiation-induced translational control of gene expression and thus the cellular radioresponse [12]. 

HCC is the second leading cause of cancer-related deaths in China. Transcriptomic analyses have revealed that splicing aberrations occur in HCC tumor tissues and are correlated with HCC patient survival [13]. Currently, SBRT shows increasing local control and therapeutic effects on primary and metastatic HCC, partly because of the radiation dose dependence of HCC [2]. The cassette exons in irradiated cells are the major AS events, which are mostly associated with DNA damage response and apoptosis [14]. Together, AS is a key component of cellular response to radiation, and splicing factors are believed to play roles in mediating and regulating AS events. PRMT5, as a tumor-promoting factor, is overexpressed in numerous cancers, including HCC, by participating in processes such as viral carcinogenesis, cell cycles, and spliceosome assembly [7,15,16,17,18]. Nevertheless, no inhibitor targeting PRMT5 has been approved for marketing to date, partly because PRMT5 deficiency has hematological toxicity [19]. Notably, we found that *PRMT5* AS occurred in both HCC patients who underwent SBRT and Huh7 cells who received IR treatment, leading to the generation of the spliced isoform *PRMT5-ISO5* (Figure 1D). Compared with full-length *PRMT5*, *PRMT5-ISO5* lacks exon 3 and partial exon 4 and translates the truncated protein (Figure 1A), which was reported to influence its protein methyltransferase activity and regulate genes involved in apoptosis and differentiation [8]. By observing that the elevated level of *PRMT5-ISO5* is associated with an improved poor prognosis of HCC patients (Figure 1B), we inferred that *PRMT5* splicing in response to IR probably contributed to the effect of SBRT on HCC.

### 3.2. SRSF3 and HNRNPH1 Play Opposite Roles in Regulating PRMT5 AS Induced by IR, Which Depends on Their Competitive Binding Abilities

The disturbed expression of splicing factors in cancers impairs the function of target apoptotic genes through extrinsic and intrinsic pathways, even significantly affecting responses to chemotherapy [5,20,21,22,23,24,25]. HNRNPH1, as a member of the hnRNP family, is associated with the methyltransferase function of PRMT5 [7] and is involved in the tumorigenic progression of Burkitt lymphoma and HCC [26,27,28]. In particular, HNRNPH/F can inhibit the combination of U2AF65 and the polypyrimidine tract by binding to G tracts situated upstream from exon 3 of *PRMT5* [8]. Although our work did not show evidence that IR induced a significant change in HNRNPH1 levels, we detected that HNRNPH1 interacted with MEP50 and was a substrate methylated by PRMT5 [29] (Figure S2E). Other studies have proven that HNRNPH1 participates in the splicing process with other splicing factors, such as SRSFs [30], suggesting that other potential regulators might be involved in *PRMT5* splicing in response to IR treatment. By evaluating the potential regulators among SRSFs, we found that SRSF3 was significantly downregulated after IR treatment (Figure 2K–N), indicating the possibility of SRSF3 in regulating *PRMT5* splicing. As an unfavorable prognostic predictor in HCC [28,31], the decreased expression of SRSF3 can induce p53β transcription and lead to p53-mediated cellular senescence [32]. 

Herein, we confirmed that HNRNPH1 silencing led to a *PRMT5-ISO5* decrease, while SRSF3 silencing resulted in an increase in *PRMT5-ISO5* levels (Figure 3A,B). In addition, we also found that exogenous HNRNPH1 decrease and exogenous SRSF3 increase made the switch of *PRMT5-ISO5* transcript from a higher level to a lower level (Figure 4A–D), and both regulators shared similar affinities to the binding region around the 3′ss on intron 2 and the alternative 3′ss on exon 4 (Figure 4G–H). Consequently, SRSF3 and HNRNPH1 probably have a competitive interaction on binding to *PRMT5* pre-mRNA and promoting the selection of alternative 3′ss on exon 4. Finally, we also observed a significant decrease in SRSF3 expression and inconspicuous level changes in HNRNPH1 after IR treatment (Figure 2K–N), suggesting that SRSF3 downregulation might be the major factor for the elevated level of *PRMT5-ISO5* induced by IR and that HNRNPH1 probably weakened its regulatory capacity in *PRMT5* splicing without changing expression level.

### 3.3. IR Induces the Elevated Level of PRMT5-ISO5 Which Increases Cell Radiosensitivity, Resulting in a Therapeutic Effect on Xenograft Tumors

As previously mentioned, radiation could induce AS events and change the radiosensitivity of tumor cells. Truncated PRMT5 can exhibit distinct localization and preferential control of critical genes for cell cycle arrest [8]. We assumed that the elevated level of *PRMT5-ISO5* sensitized HCC cells to IR and thus improved the radiotherapeutic effect and poor prognosis in HCC patients. In the beginning, we observed that exogenous expression of truncated PRMT5 had an inadequate effect on recovering colony-forming ability after IR treatment, which was equivalent to PRMT5 deficiency (Figure 5A). This finding supported that the increase in truncated PRMT5 expression or *PRMT5-IO5* levels could sensitize cells to IR treatment. In vivo, we also revealed tumor volume reduction, tumor cell degeneration, and necrosis in xenograft tumors after IR treatment (Figure 5C,D). Although intratumoral injection of in vivo siRNA either enhanced or slightly interfered with the antitumorigenic effect of IR, *PRMT5-ISO5* still improved the efficiency of radiotherapy in HCC cells. Despite the limitation of follow-up time after treatment, tumor growth inhibition and cell necrosis in the treated groups supported that *PRMT5-ISO5* induced by IR was effective in improving poor prognosis in HCC patients.

### 3.4. Liver-Specific Knockout of Prmt5 Inhibits Hepatocarcinogenesis

Accumulating studies indicate that PRMT5 might be a potential therapeutic target in HCC, but the limitation of in vivo studies is the usage of HCC cell lines or xenograft models by passing over the efficacy in the remedy of spontaneous HCC [6]. Currently, transposon-based liver-specific delivery through HTVi has become the perfect technique for the establishment of HCC to study potential oncogenic genes [15,33,34]. Regarding the molecular mechanism, for instance, activation of the AKT signaling pathway induces cell proliferation, lipogenesis, and tumor development in HCC [35]; hepatic expression of Nras(G12V) triggers oncogene-induced senescence [36]; coexpression of hMet and mutant-β-catenin activates hMet and Wnt pathways [37], thus leading to notable HCC in mice. Akt/N-Ras-based HTVi technology is reported to rapidly induce HCC formation accompanied by fatty change lesions [38] and microenvironment change [39], and PRMT5 is involved in regulating the expression of lipogenic genes [40] and promoting cell proliferation [7]. By recapitulating aberrant lipid metabolism involved in HCC development, we observed that liver-specific Prmt5 KO remarkably decelerated the tumorigenesis of Akt/N-Ras-transformed hepatocytes such as vacuolar denaturation, inflammation, and intracellular lipid accumulation (Figure 6C,D). In addition, recent studies also showed that the inhibition of PRMT5 with immune checkpoint therapy diminished the growth of murine melanoma tumors and enhanced therapeutic efficacy [41], and suppressive macrophage infiltrates and exhausted-like phenotypes and tumor-associated antigen-specific CD8^+^ T cells were present during Akt/N-Ras-derived HCC progression [34,38,42]. The latest research implicates that the PISK/AKT pathway is involved in radiotherapy resistance in HCC patients, and ablation of PDK1 function might improve radiosensitivity and is associated with deactivated PI3K/AKT/mTOR signaling [43]. In addition, PRMT5 methylates AKT1 on Arg 15 in the PH domain, promoting AKT1 translocation to the plasma membrane and subsequent phosphorylation at Thr308 and Ser473 in neuroblastoma [44]. In our work, we confirmed that Prmt5 deficiency reduced the phosphorylation of Akt at Ser473 and inhibited the progression of HCC (Figure 6E), suggesting that Prmt5 depletion inhibits Akt/N-Ras-derived hepatocarcinogenesis, which might partly be due to the dysfunction of methylating Akt1 for oncogenic activation and improving immune dysregulation. Based on the results the exogenous expression of truncated PRMT5 sensitized Huh7 cells to IR, which was equivalent to PRMT5 deficiency. It is important to further investigate whether *PRMT5-ISO5* acts analogously to Prmt5 depletion in primary HCC. Nevertheless, our study provides a suitable preclinical model for further elucidation of the function of *PRMT5-ISO5* in HCC progression and therapies in vivo.

## 4. Materials and Methods

### 4.1. Patients

The inclusive criteria for HCC patients were mentioned previously [45]. All patients provided informed written consent, and all research and related activities involving human subjects were approved by the Ethics Committee of the first and second hospitals affiliated with Jilin University. RNA sequencing (RNA-seq) and analysis were completed by Novogene Biotech.

The liver hepatocellular carcinoma (LIHC) patient datasets were derived from the TSVdb TCGA Splicing Variants Database (http://www.tsvdb.com/plot.html, accessed on 14 September 2021). The single-cell RNA sequencing (scRNA-seq) dataset (GSE149614) was derived from the Gene Expression Omnibus database (https://www.ncbi.nlm.nih.gov/geo/, accessed on 8 April 2022).

### 4.2. Cell Culture

HEK293T cells and three human HCC cell lines (Huh7, HepG2, and MHCC-97H cells) were cultured in Dulbecco’s modified Eagle’s medium (DMEM) supplemented with 10% fetal bovine serum (FBS, Gibco, Carlsbad, CA, USA) and penicillin/streptomycin at 37 °C with 5% CO_2_.

### 4.3. IR Treatment

The X-Rad 320 Biological Irradiator (Precision X-Ray, Greenville, SC, USA) was used at a dose rate of 300 cGy/min.

### 4.4. Plasmid Construction and siRNAs

The *PRMT5*-minigene, a segment of PRMT5 including the region from exon 1 to exon 4, was amplified by polymerase chain reaction (PCR) from genomic DNA. The human HNRNPH1 and SRSF3 open reading frames (ORFs) were amplified by RT-PCR. The primers are listed in Supplementary Table S1. Next, the PCR products were gel-purified with the Gel Extraction Kit (Takara, Beijing, China) and then in-fusion cloned into the pcDNA 3.1-FLAG vector, which had been digested by BamHI and XhoI restriction endonucleases (NEB, Beijing, China) using the ExonArt Seamless Cloning and Assembly Kit (Exongen, Chengdu, China). The full-length PRMT5 (Myc-PRMT5L) and short-length PRMT5 ( Myc-PRMT5S, which was translated from *PRMT5-ISO5*) plasmids were generous gifts from Prof. Jiuyong Xie (Department of Physiology & Pathophysiology, University of Manitoba) as described [46].

The normal siRNA and In vivo siRNA (2′ O-methyl + 5′ cholesterol-modified) against HNRNPH1 or SRSF3 and nonsense siRNA (RiboBio, Guangzhou, China) were dissolved in rNase-free water for cell transfection or phosphate buffered saline (5 nmol siRNA with 50 μL PBS) for intratumoral injection. The sequences of si-HNRNPH1 and si-SRSF3 are shown in Supplementary Table S1 [47,48].

### 4.5. Cell Transfection

RNA interference and construct overexpression were carried out in 6-well plates using Lipofectamine 2000 (Invitrogen, Carlsbad, CA, USA) with 5 μL siRNA (final concentration of 50 nM) or 2.5 μg plasmid. For the cotransfection assay, 2 μg *PRMT5*-minigene plasmid was co-transfected with 4 μL siRNA (final concentration of 40 nM) or 2 μg plasmid in PRMT5 KO cells cultivated in 6-well plates. To investigate the HNRNPH1 and SRSF3 interaction, 4 μg *PRMT5*-minigene plasmid was co-transfected with a total of 4 μg plasmid mixture (pcDNA3.1-FLAG-HNRNPH1 plus pcDNA3.1-FLAG-SRSF3) in PRMT5 KO cells grown in 60 mm Petri dishes. The cells were harvested 48 h post-transfection. The levels of *PRMT5-ISO5* were determined by RT-qPCR, and the expression of HNRNPH1 and SRSF3 was detected by western blotting.

### 4.6. Generation of PRMT5 Knockout Clones

Cas9 lentivirus and *PRMT5*-sgRNA lentivirus were designed and generated in HEK 293T cell lines by cotransfection of psPAX2, pMD2.G, and Cas9 or *PRMT5*-sgRNA plasmids at a 4:3:1 ratio. A total of 2.5 μg plasmids were mixed with Lipofectamine 2000 reagent (Thermo Fisher Scientific, Waltham, MA, USA) in a well of 6-well plates when HEK 293T cells were 60–70% confluent. Huh7 and MHCC-97H cell lines were infected with Cas9 lentivirus for 24 h and then treated with hygromycin for 7–10 d. Subsequently, Cas9-positive cells were infected with *PRMT5*-sgRNA lentivirus for another 24 h and then treated with puromycin for 5–7 d. Furthermore, serial dilution was performed to generate single clones of stable PRMT5 knockout (KO) cell lines. Finally, all clones were evaluated for PRMT5 KO efficiency by western blotting.

### 4.7. Colony Formation Assay

PRMT5 KO Huh7 clones were rescued with either Myc-PRMT5L or Myc-PRMT5S plasmids separately. The transfected cells (48 h post-transfection), Cas9-positive Huh7 cells, and PRMT5 KO Huh7 clones were seeded at a density of 1 × 10^3^, 2 × 10^3^, 3 × 10^3^, 4 × 10^3^, or 6 × 10^3^ cells per well in 6-well plates for another 24 h. Subsequently, the cells with different starting cell numbers were untreated (0 Gy) or treated with IR of 2 Gy, 4 Gy, 6 Gy, and 8 Gy and then incubated with DMEM for 10 days at 37 °C. Finally, paraformaldehyde (Solarbio, Beijing, China) was used for fixation, and crystal violet (Solarbio, China) was employed to stain the forming colonies.

### 4.8. Reverse Transcription-Quantitative PCR and Western Blotting

Total RNA was extracted using RNAiso Plus (Takara, China) according to the manufacturer’s protocol. Quantitative PCR analysis of *PRMT5-ISO5* was carried out with 10 μL TB Green™ Premix Ex Taq™ II (Takara, China) by the QuantStudio 3 Real-Time PCR System (Applied Biosystems, Waltham, MA, USA). The primers for RT-qPCR are shown in Supplementary Table S1.

Proteins extracted from cells and tissues were measured and heat denatured. Equal amounts of proteins were separated using 10% or 12% SDS-PAGE and transferred to PVDF membranes (Millipore, Boston, MA, USA). After saturating, the membranes were incubated with anti-PRMT5 (ab109451, Abcam, Boston, MA, USA), anti-HNRNPH1 (ab10374, Abcam, Boston, MA, USA), anti-SRSF3 (ab198291, Abcam, Boston, MA, USA), anti-AKT1 (75692S, CST, Boston, MA, USA), anti-phospho-AKT (Ser473) (4060S, CST, Boston, MA, USA), anti-SDMA (13222S, CST, Boston, MA, USA) or anti-GAPDH (60004-1-Ig, Proteintech, Shanghai, China), and subsequently incubated with HRP-conjugated secondary antibodies (Biosharp, Guangzhou, China). Finally, the membranes were detected using Pierce™ ECL Western Blotting Substrate (Thermo Fisher Scientific, Waltham, MA, USA) and visualized with ChemiScope 6100 (CliNX Science Instruments, China).

### 4.9. RNA Immunoprecipitation

Cells were transfected with FLAG-tagged SRSF3 or HNRNPH1 using Lipofectamine 2000 reagent (Thermo Fisher Scientific, Waltham, MA, USA). Forty-eight hours post-transfection, the cells were washed and collected in lysis buffer (10 mM Tris-HCl (pH 7.5), 100 mM NaCl, 5 mM MgCl2, 0.5% NP-40, 1% Triton X-100) containing EDTA-free Protease Inhibitor Cocktail (Roche, Basel, Switzerland), 1 mM DTT (Thermo Fisher Scientific, Waltham, MA, USA) and 200 units/mL RNase OUT (Invitrogen, Carlsbad, CA, USA). Then, 10% of the cell lysate was taken as the “input”, and the remains were divided into equal amounts and incubated overnight at 4 °C with anti-FLAG magnetic beads (Bimake, Suzhou, China) or preprepared IgG-protein A/G magnetic beads (Bimake, Suzhou, China). Subsequently, all the mixtures were washed 6 times with 1×NT-2 buffer (50 mM Tris-HCl (pH 7.5), 150 mM NaCl, 1 mM MgCl2, 0.05% NP-40) using the DynaMagTM-2 Magnet (Invitrogen, Carlsbad, CA, USA). Next, the washed samples were digested at 55 °C for 30 min with proteinase K digestion buffer (1 × NT-2 buffer containing 1% SDS and 1.2 mg/mL proteinase K). Finally, immunoprecipitated RNA was extracted using RNAiso Plus (Takara, Dalian, Shanghai, China). Fold enrichment of the target region was determined after normalization to the input and compared with the IgG control. The primers for RT-qPCR are shown in Supplementary Table S1.

### 4.10. Xenograft Model and Treatments

Male BALB/c nude mice (4–5 weeks old, Zhejiang Vital River Laboratory Animal Technology, Zhejiang, China) were kept under pathogen-free conditions with a 12 h light/dark cycle and had free access to water and food. After acclimation for a week, 1 × 10^7^ Huh7 cells were mixed with Matrigel Basement Membrane Matrix (BD, Franklin Lakes, NJ, USA) at 4 °C and then injected into the right flanks of nude mice. When the tumors were visible (160 mm^3^ in volume), the mice were divided into 4 groups and treated with (1) 15 Gy; (2) 15 Gy and intratumoral injection of si-HNRNPH1 (5 nmol for each); (3) 15 Gy and intratumoral injection of si-SRSF3 (5 nmol for each) [49]; and (4) untreated control. All animal care and experimental procedures were carried out in accordance with protocols approved by the Wenzhou Medical University Institutional Animal Care and Use Committee.

### 4.11. Hydrodynamic Tail-Vein Injection and Prmt5 Conditional Knockout Experiments

Male Prmt5^flox/flox^-Alb-CreERT2 mice with a C57BL/6J background (4–5 weeks old) were generated and purchased from Cyagen Biosciences. The mice were housed under pathogen-free conditions with a 12 h light/dark cycle and allowed free access to water and food. pCMV(CAT)T7-SB100 and pT3-myr-AKT-HA were purchased from Fenghui Biotechnology (Addgene plasmids #34879 and #31789), and pT3-EF1α-NRasV12 (RPT-ZL 2101C2) was purchased from RiboBio. After acclimation for a week, hydrodynamic tail-vein injection (HTVi) of a volume equivalent to 10% body weight of endotoxin-free plasmids (10 μg of pCMV(CAT)T7-SB100, 10 μg of pT3-myr-AKT-HA, and 10 μg of pT3-EF1α-NRasV12 for each mouse [39,50,51]) dissolved in PBS was given to Prmt5flox/flox-Alb-CreERT2 mice within 7 s [36,38]. Registering morbidity (white spots presented on the liver) according to prespecified criteria, the mice were randomly divided into conditional KO (CKO) cohorts and non-CKO controls at approximately 7.4 weeks post-HTVi. The mice from the CKO group received a continuous intraperitoneal injection of 100 μL tamoxifen (predissolved in corn oil at 10 mg/mL, 40 mg/kg per mouse, Sigma-Aldrich, St. Louis, MO, USA) for 7 days. The mice were euthanized, and liver (tumor) tissues were collected for further analyses. All animal care and experimental procedures were carried out in accordance with protocols approved by the Wenzhou Medical University Institutional Animal Care and Use Committee.

### 4.12. Tumor Growth and Histochemistry Assay

The xenograft tumor volume was measured every 2 days and calculated as follows: volume = longest tumor diameter × (shortest tumor diameter)^2^/2. At the end of the experiment, mice were sacrificed by euthanasia, tumor tissues were excised and weighed, images were captured, and immunohistochemistry was performed.

Liver (tumor) tissue sections were paraffin-embedded, deparaffinized, and rehydrated, followed by hematoxylin and eosin (H&E) staining. For detection of lipid droplets, liver tissue sections were incubated with oil red O dye and washed with 60% isopropanol. Finally, the images were captured and evaluated.

### 4.13. Statistical Analysis

The LIHC patient datasets were analyzed using the Kruskal–Wallis test and Kaplan–Meier curves (log-rank tests) in R software. The scRNA-seq data were preprocessed by using the Seurat package. The harmony package and t-distributed stochastic neighbor embedding (t-SNE) algorithm were used to perform normalization, dimensionality reduction, and cluster classification. Gene Ontology (GO) analysis was conducted with clusterProfiler packages. GEPIA2 [52] was used for expression correlation and survival analysis.

Each experiment was performed in triplicate and the representative data from one experiment are shown. Statistical analysis for biological assays was performed by GraphPad Prism 7 software using one-way or two-way ANOVA and a two-tailed unpaired *t*-test, as appropriate. All data are presented as the mean ± S.D. Differences were considered statistically significant if *p <* 0.05, * *p* < 0.05, ** *p* < 0.01, *** *p* < 0.001, **** *p* < 0.0001.

## 5. Conclusions

**Figure 7:**
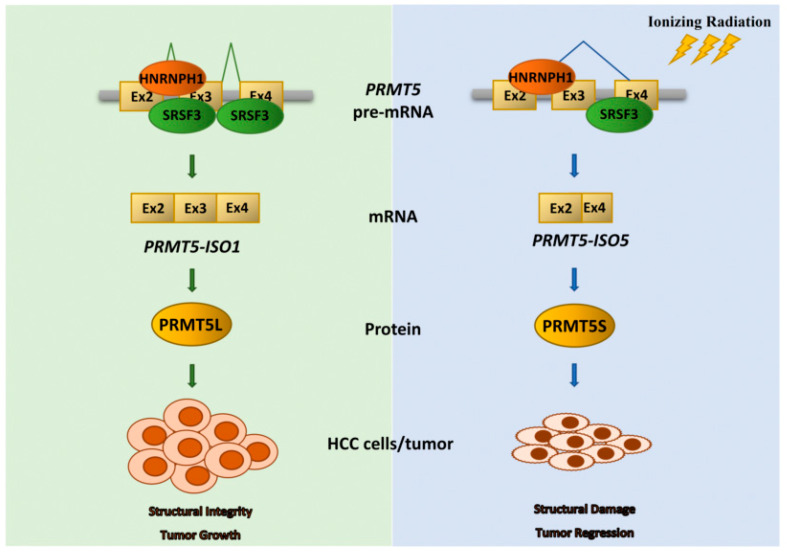
A schematic diagram illustrating SRSF3 downregulation regulates the increase in *PRMT5-ISO5* and plays a positive role in radiotherapy of HCC. Normally, canonical *PRMT5-ISO1* is the main isoform that translates into full-length PRMT5 (PRMT5L) and promotes HCC development (**left**). After IR treatment, SRSF3 (S3) downregulation breaks its balance with HNRNPH1 (H1) in binding to PRMT5 pre-mRNA and generates *PRMT5-ISO5*. The increased *PRMT5-ISO5* translates into the short-length protein (PRMT5S), which enhances cell radiosensitivity and promotes tumor regression of HCC (**right**).

In conclusion, we provide preclinical evidence that *PRMT5-ISO5* might be a putative enhancer of radiosensitivity and a potential therapeutic approach for HCC patients. Briefly, IR induces an increase in *PRMT5-ISO5* which improves poor prognosis. SRSF3 and HNRNPH1 antagonistically regulate *PRMT5* AS by virtue of their competitive binding to *PRMT5* pre-mRNA and promoting the choice of alternative 3′-ss on exon 4 rather than 3′-ss on intron 2. Notably, IR decreases SRSF3 expression and results in an increase in *PRMT5-ISO5* levels, which enhances cell radiosensitivity and xenograft tumor regression (Figure 7). Finally, Akt/N-Ras-derived spontaneous HCC with a liver-specific Prmt5 KO model indicates the malignant effects of Prmt5 on tumorigenesis and provides a rationale to investigate the role of the specific spliced isoform in primary HCC radiotherapy.

## Supplementary Materials

The following supporting information can be downloaded at: https://www.mdpi.com/article/10.3390/ijms232314832/s1.
