# Supplementary material for: SRSF3 and HNRNPH1 Regulate Radiation-Induced Alternative Splicing of Protein Arginine Methyltransferase 5 in Hepatocellular Carcinoma
Source: Int J Mol Sci. 2022 Nov 27;23(23):14832. doi: 10.3390/ijms232314832 (PMC9738276; doi:10.3390/ijms232314832)
Supplement: Supplementary file 1 [file ijms-23-14832-s001.zip › ijms-1997332-final-supplementary.pdf]

## Supplementary Materials:

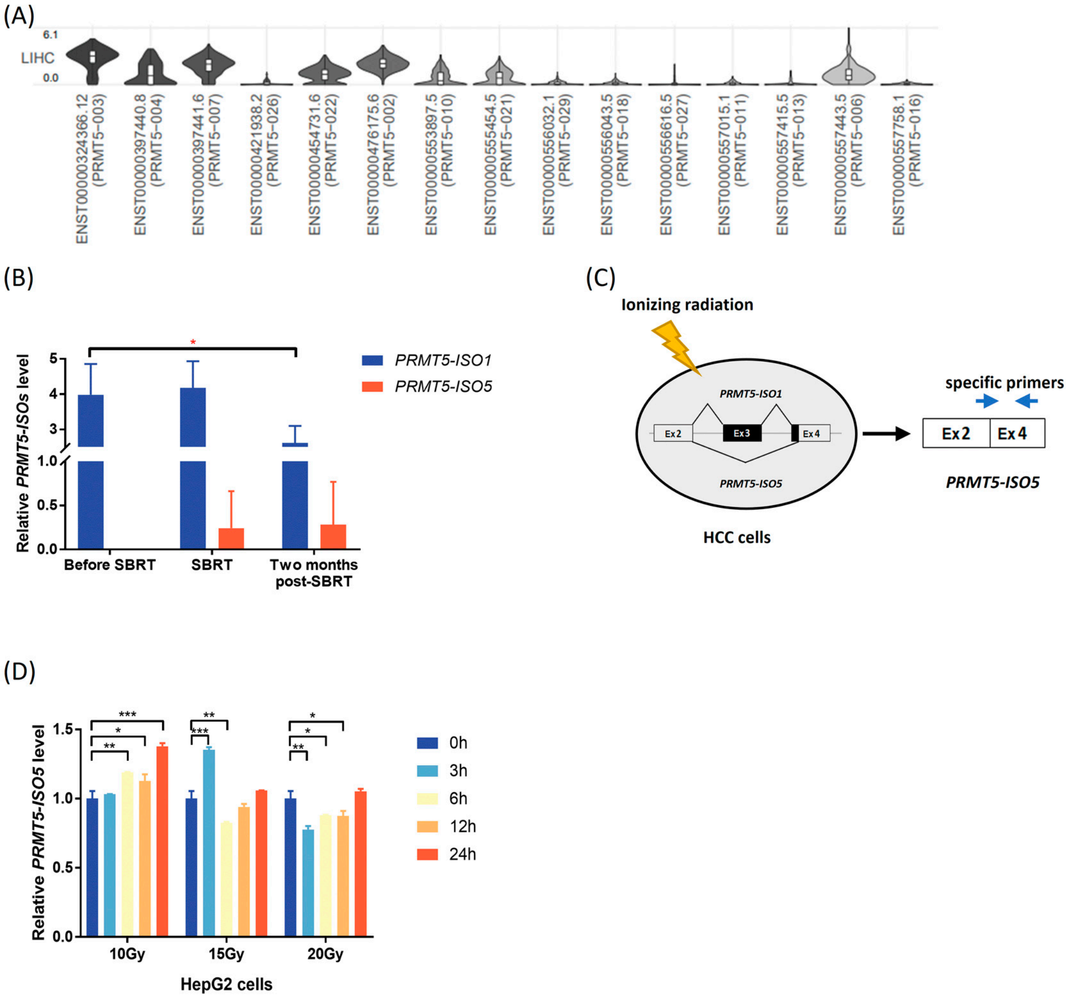


**Figure S1. IR induced *PRMT5* splicing in HCC.** (A) The expression levels of *PRMT5* isoforms in LIHC patients. PRMT5-003: *PRMT5-ISO1*; PRMT5-010: *PRMT5-ISO5*. (B) The transcriptional level of *PRMT5-ISO1* and *PRMT5-ISO5* in HCC patients after SBRT (n=3). (C) The procedure of IR-induced *PRMT5-ISO5* production and target region for *PRMT5-ISO5* detection. (D) The transcriptional level of *PRMT5-ISO5* induced by IR in HepG2 cells. $P < 0.05$, $^{**}P < 0.01$, $^{***}P < 0.001$.

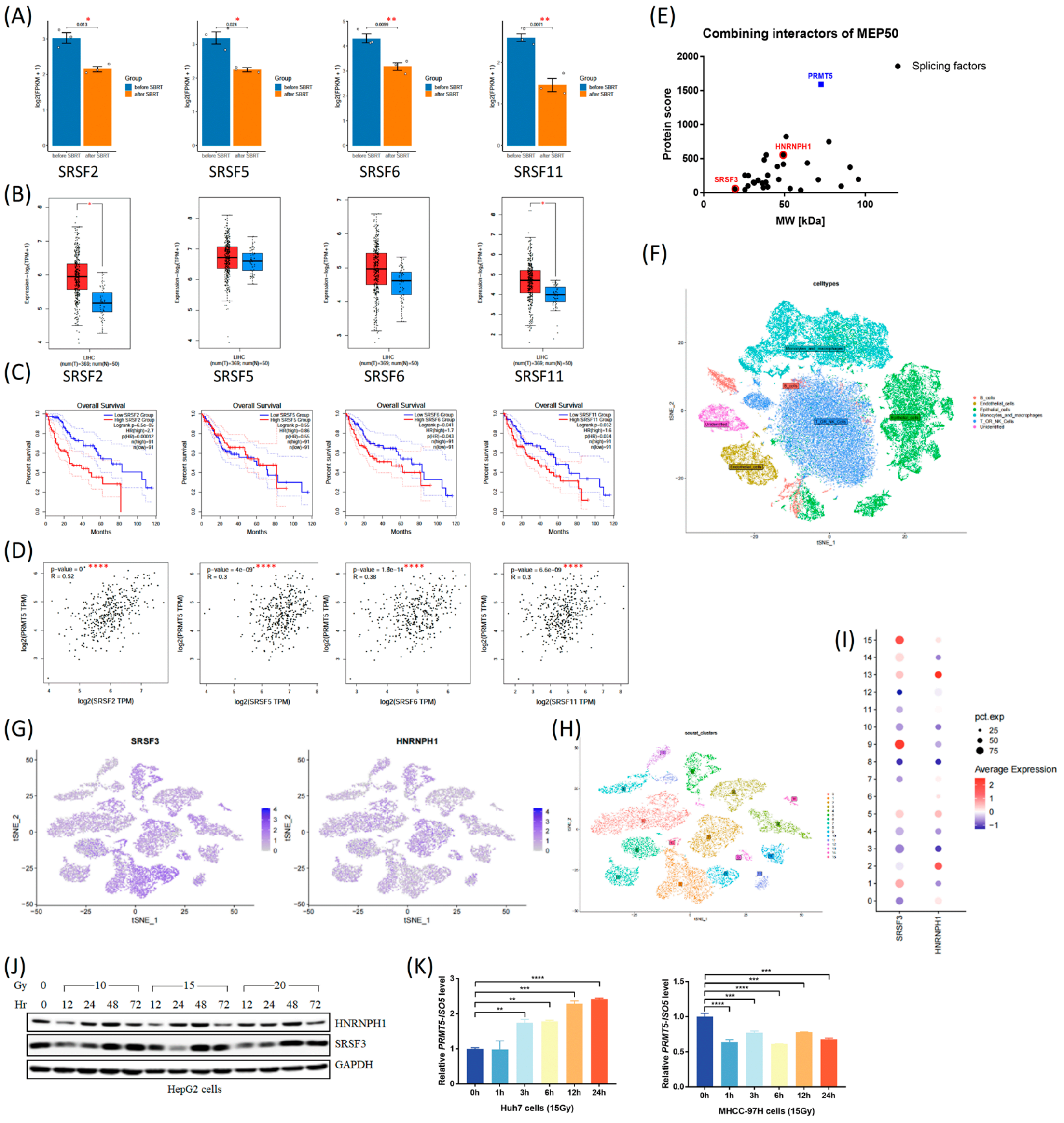


**Figure S2. IR-induced *PRMT5-ISO5* is regulated by SRSF3 and HNRNPH1.** (A) The transcriptional level of SRSF2, SRSF5, SRSF6, and SRSF11 in HCC patients received SBRT (n=3). (B-D) Bioinformatics analysis of LIHC patients and normal controls based on GEPIA2. (B) The differential expression of SRSF2, SRSF5, SRSF6, and SRSF11. (C) The overall survival analysis diagram of SRSF2, SRSF5, SRSF6, and SRSF11. (D) The correlation analysis of PRMT5 and SRSF2, SRSF5, SRSF6, or SRSF11. $^{*}P < 0.05$, $^{**}P < 0.01$, $^{****}P < 0.0001$. (E) Splicing factors interacting with methylosome protein 50 (a component of PRMT5) from immunoprecipitation-mass spectrometric analysis. (F) t-SNE of the different cell clusters and the cell types identified according to subtype markers from scRNA-seq datasets (GSE149614). (G-I) Combined analysis of the expression distribution and intercellular communication of SRSF3 and HNRNPH1 in identified cell clusters from scRNA-seq datasets (GSE149614). (J) The expression level of HNRNPH1 and SRSF3 induced by IR in HepG2 cells. (K) The transcriptional level of *PRMT5-ISO5* induced by IR within 24 h in Huh7 and MHCC-97H cells. $P < 0.05$, $^{**}P < 0.01$, $^{***}P < 0.001$.

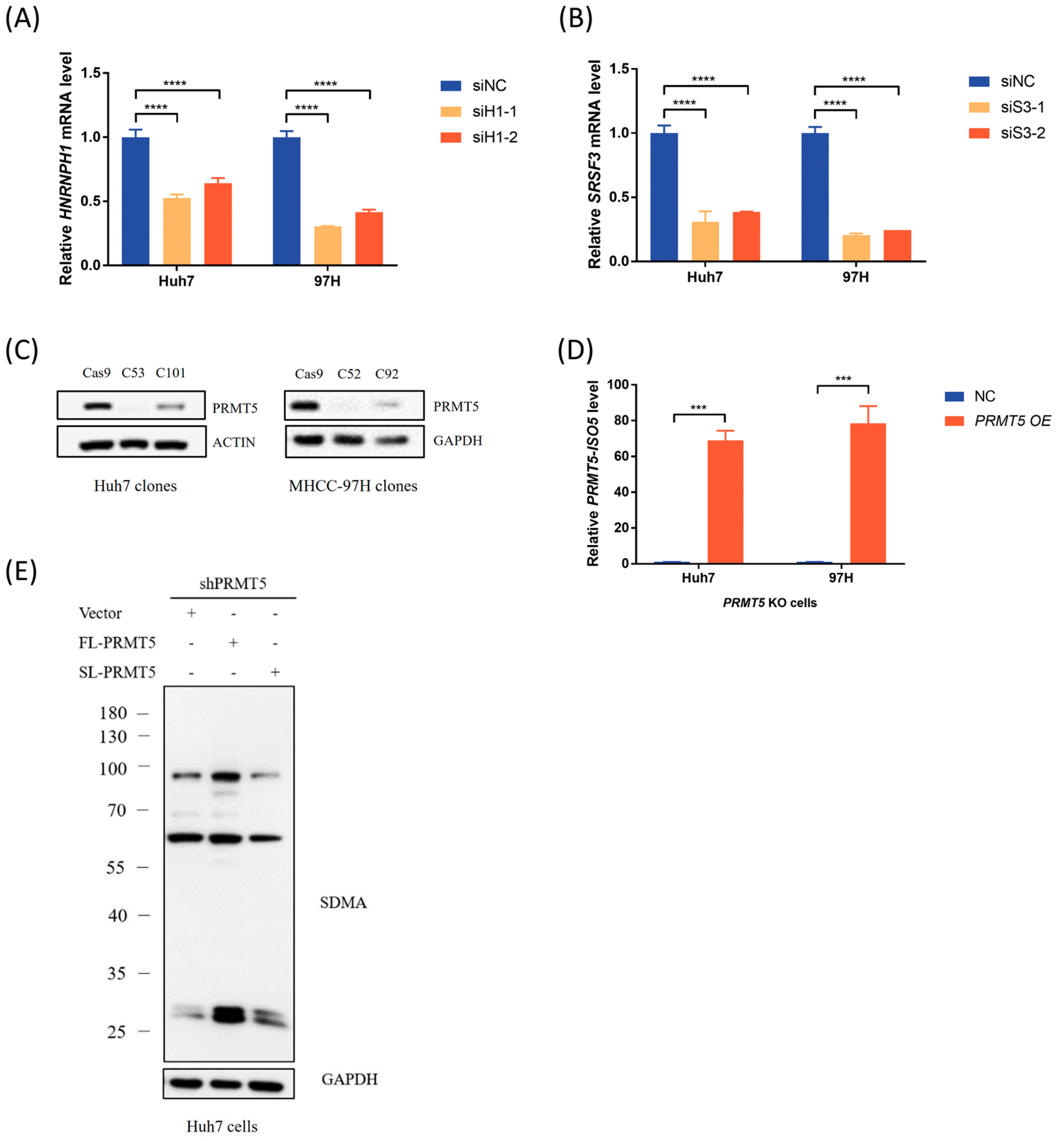


**Figure S3. SRSF3 and HNRNPH1 play opposite roles in *PRMT5-ISO5* production induced by IR.** (A-B) The efficiencies of HNRNPH1 silencing or SRSF3 depletion detected by RT-qPCR. siH1-1/2: si-*HNRNPH1*-1/2, siS3-1/2: si-*SRSF3*-1/2; siNC: nonsense siRNA. $^{****}P < 0.0001$. (C) Representative *PRMT5* KO clones (C52, C53, C92 and C101) were confirmed by PRMT5 detection compared with Cas9 positive controls (NC). (D) The efficiencies of the *PRMT5*-minigene overexpression detected using RT–qPCR. NC: PRMT5 KO; PRMT5 OE: PRMT5-minigene overexpression. $^{***}P < 0.001$. (E) The level of SDMA modification in PRMT5 knockdown Huh7 cells with full-length PRMT5 (FL-PRMT5) or truncated PRMT5 (SL-PRMT5) rescue.

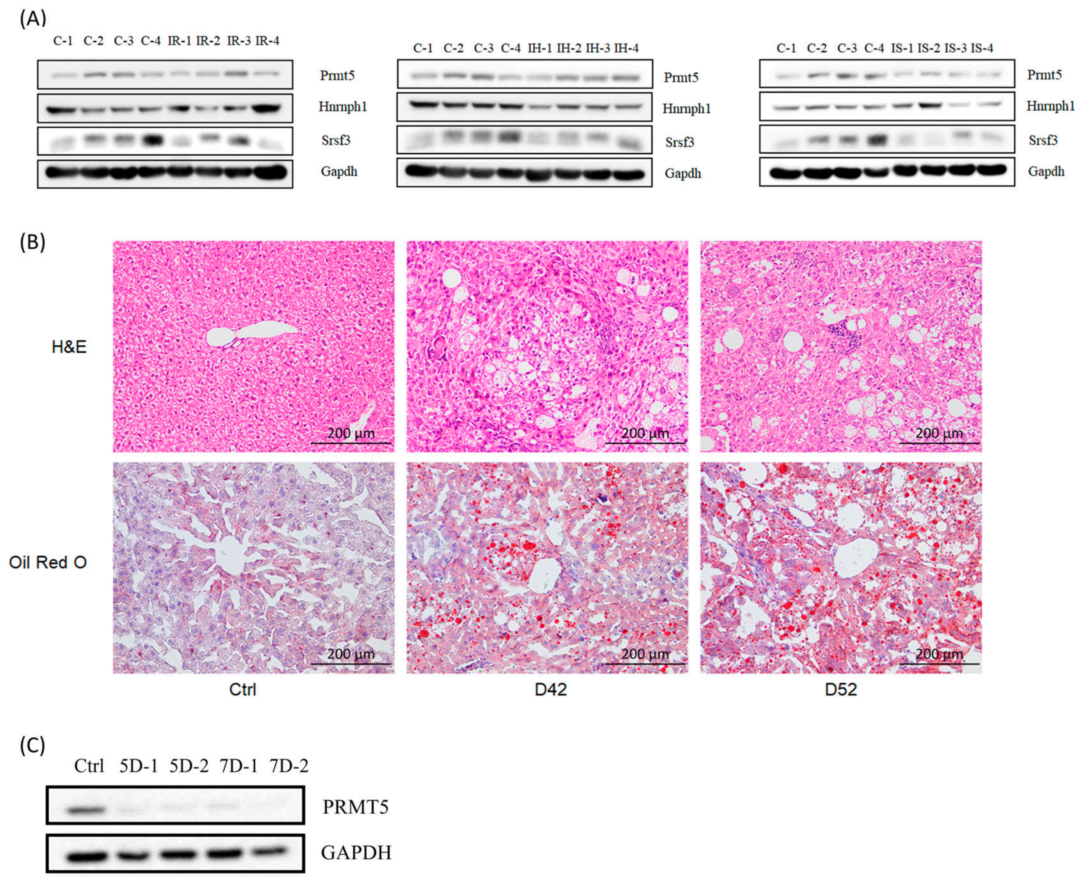


**Figure S4. Liver-specific Prmt5 deficiency inhibits primary HCC progression.** (A) The expression level of PRMT5, HNRNPH1 and SRSF3 of HCC xenograft tumors at the $8^{\text{th}}$ day post-treatment (n=4 in each group). (B) The morphological change of liver tissue or tumor at the $62^{\text{nd}}$ day post-HTVi treatment (200×). (C) The expression level of PRMT5 in the liver from *Prmt5*$^{\text{flox/flox}}$-Alb-CreERT2 mice with or without continuous intraperitoneal injection of tamoxifen (10 mg/mL). 5D-1/2: $1^{\text{st}}$ day and $30^{\text{th}}$ day after continuous intraperitoneal injection of tamoxifen for 5 days; 7D-1/2: $1^{\text{st}}$ day and $30^{\text{th}}$ day after continuous intraperitoneal injection of tamoxifen for 7 days.

**Table S1. List of primer sequences used in the study**

| | |
|---|---|
| **Primers of cloning** | |
| *PRMT5*-minigene | F: 5’-gataagagcccgggcggatccATGGCGGCGATGGCGGTC-3’ |
| | R: 5’-aacgggccctctagactcgagCATGGAAGAGTGATGGCCAGTG-3’ |
| *HNRNPH1* | F: 5’- gataagagcccgggcggatccATGGCGGCGATGGCGGTC-3’ |
| | R: 5’-aacgggccctctagactcgagCATGGAAGAGTGATGGCCAGTG-3’ |
| *SRSF3* | F: 5’-gataagagcccgggcggatccATGCATCGTGATTCCTGTCCA-3’ |
| | R: 5’-aacgggccctctagactcgagCTATTTCCTTTCATTTGACCTAGATCG-3’ |
| **Primers of RT–qPCR** | |
| *PRMT5-ISO5* | F: 5’-GGAAGGGCTTTCCTGCTG-3’ |
| | R: 5’-GGTACCCGCATCCAGAAC-3’ |
| *HNRNPH1* | F:5’- ATTCAAAATGGGGCTCAAGGTAT-3’ |

| | |
|---|---|
| | R:5’-GTGTCAGGACTATTTGGACCAG-3’ |
| *SRSF3* | F: 5’-ATGGAAGAACACTATGTGGCTG-3’ |
| | R: 5’-GGGACGGCTTGTGATTTCTCT-3’ |
| *PRMT5* (total) | F: 5’-CAGGAACCTGCTAAGAATCG-3’ |
| | R: 5’-GCCAGTGTGGATGTGGTTG-3’ |
| *β-actin* | F: 5’-CACCAACTGGGACGACAT-3’ |
| | R: 5’-ACAGCCTGGATAGCAACG-3’ |
| **Primers of RNA immunoprecipitation RT–qPCR assay** | |
| Intron 2-Exon 3 | F:5’-ACCTTTGTCTCTCATTTCTACTCTT-3’ |
| | R:5’-GCTTTCCCACAATTAGCGTATTC-3’ |
| Intron 3 | F:5’-AAGACTGTTTGACTTCTCTGCT-3’ |
| | R:5’-CAGCACCCAGCCTAATAGC-3’ |
| Intron 3- Exon 4a | F:5’-TCCTCTTCATCTCCGTTCCA’ |
| | R:5’-GGCCAGGTTGGTGTTATCTT-3’ |
| Exon 4 | F:5’-GAGTTTTGACCAACCACATCC-3’ |
| | R:5’-GCCCGCCAATACTGATACTT-3’ |
| **Sequences of siRNAs** | |
| *si-HNRNPH1-1* | 5'-GGAAGAAAUUGUUCAGUUCtt-3’[1] |
| *si-HNRNPH1-2* | 5'-AGCUGAAGUUAGAACUCAUUA-3' |
| *si-SRSF3-1* | 5'-GCAACAAGACGGAAUUGGAtt-3'[2] |
| *si-SRSF3-2* | 5'-UGGAACUGUCGAAUGGUGAAA-3' |
| *si-NC* | siN0000001-1-5 (RiboBio) |
| In vivo *si-HNRNPH1* | 5'-GGAAGAAAUUGUUCAGUUCtt-3’ (2OMe+5Chol) |
| In vivo *si-SRSF3* | 5'-GCAACAAGACGGAAUUGGAtt-3' (2OMe+5Chol) |
| In vivo *si-NC* | siN0000005-4-5 (2OMe+5Chol, RiboBio) |
| **Sequences of PRMT5 sgRNA in CRISPR/Cas9 system** | |
| sgRNA#1: | TCGCGATTATTGTCTGGGAGTGG |
| sgRNA#2: | GAACCCAAGATAGTCTAATCTGG |
| **Primers of *PRMT5* KO sequencing** | |
| sgRNA-1 | F:5’-TTGCGTCCCCGAAATAGCTG-3’ |
| | R: 5’-ACAGCCATGGGACATATGAGT-3’ |
| sgRNA-2 | F: 5’-CTCTGTGCCCCTAGCAAATGAA-3’ |
| | R: 5’-CTGCTTCCAACTACTGTCATGGA-3’ |

## References


1. Nazim, M.; Masuda, A.; Rahman, M.A.; Nasrin, F.; Takeda, J.I.; Ohe, K.; Ohkawara, B.; Ito, M,; Ohno, K. Competitive regulation of alternative splicing and alternative polyadenylation by hnRNP H and CstF64 determines acetylcholinesterase isoforms. Nucleic Acids Res. 2017, 45, 1455–1468. . https://doi.org/10.1093/nar/gkw823.

2. Chen, J.; Crutchley, J.; Zhang, D.; Owzar, K.; Kastan, M.B. Identification of a DNA Damage-Induced Alternative Splicing Pathway That Regulates p53 and Cellular Senescence Markers. Cancer Discov. 2017, 7, 766–781. https://doi.org/10.1158/2159-8290.CD-16-0908
